# User Needs in the Development of a Health App Ecosystem for Self-Management of Cystic Fibrosis: User-Centered Development Approach

**DOI:** 10.2196/mhealth.8236

**Published:** 2018-05-08

**Authors:** Jacqueline Floch, Annabel Zettl, Lena Fricke, Tina Weisser, Lisbet Grut, Thomas Vilarinho, Erlend Stav, Antonio Ascolese, Cornelia Schauber

**Affiliations:** ^1^ SINTEF Trondheim Norway; ^2^ YOUSE GmbH München Germany; ^3^ SINTEF Oslo Norway; ^4^ imaginary srl Milano Italy

**Keywords:** self-management, cystic fibrosis, mobile health, user centered design

## Abstract

**Background:**

Digital self-management in cystic fibrosis (CF) is foreseen as a means toward better understanding of the disease and its treatment and better adherence to the treatment. Mobile apps hold the potential to provide access to information, motivate, and strengthen compliance. However, to deliver high-quality apps, the development should be based on thorough knowledge about user needs. Empirical research on the user-centered development of mobile apps for health care is, however, still limited.

**Objective:**

The aim of this research is to develop and evaluate an app ecosystem for self-management in CF. It targets not only those directly affected by CF but also parents and health care professionals involved in the treatment. This paper covers the first step of the design process that aims to analyze the context and the user requirements. The primary research question is as follows: what digital support has the potential to usefully support persons with CF and their caregivers in the CF care? To answer this question, we address two preliminary questions: what important factors in everyday life affect the care of persons with CF? and how is the CF care delivered today and what are the limitations of CF care services?

**Methods:**

The overall research adopts a user-centered design approach in which future users are involved in the development process from the very beginning to ensure that the apps developed best suit the potential users. The research presented in the paper follows an interpretative case study research strategy seeking to understand the concerns and needs of persons with CF and their caregivers. Data were collected through semistructured qualitative interviews involving 74 participants in seven European countries and from internet forums.

**Results:**

The results of the analysis phase show a strong need for individuality of the digital support, as well as for its adaptability to different contexts. The paper presents the concerns and needs of the participants in the study and extracts a set of relevant features for a self-management app ecosystem. Education, enzyme dosage calculation, nutrition management, treatment organization, health diary, treatment follow-up, practical guidelines for treatment, communication with doctors, and communication with peers are foreseen as useful features.

**Conclusions:**

The results indicate the readiness for self-management in the CF care even in countries that provide well-functioning health care services for CF care. The large diversity of user requirements identified reflects the crucial role user integration plays in developing apps for a chronic condition such as CF. The need for personalization stemming from the individuality of the patients and the need for communication with health care professionals support the idea of an app ecosystem for the self-management of CF.

## Introduction

### Background

The market for wearables and mobile apps for monitoring health data and receiving information and guidelines on health-related issues is growing. Half of the participants in recent user surveys would use an app to manage their health, or had already downloaded one [1,2]. Health management through apps may be particularly beneficial for persons with chronic conditions who often require daily care and sustained self-management. In fact, different reviews [3,4] have observed a positive effect of the use of information and communication technology (ICT) for facilitating self-management of chronic diseases. At the same time, the number of persons living with a chronic health condition is increasing across countries [5,6], and they are responsible for most deaths in the world [7]. This puts health services under pressure. Thus, the overall need to promote self-management in health care is strong.

Self-management is seen as a means to better understand and cope with a disease and its treatment, for example, by supporting behavior change and treatment compliance [8]. Effectively pursued self-management may contribute to increased life expectancy and a better quality of life (QoL). One way to support self-management is to increase the person’s knowledge about the condition and to foster perceived self-efficacy [9]. For successful engagement, patients need to feel empowered and enabled to participate in the management of their health [10].

In this paper, we present a case study aiming at understanding the concerns of persons affected by cystic fibrosis (CF) and their needs, and we derive implications for the design of an app ecosystem for self-management of CF. CF is a congenital, chronic metabolic disorder that affects the digestive and respiratory tracts resulting in generalized malnutrition and chronic respiratory infections. There is still no cure for the disease. CF is a lifelong condition that often begins to affect the daily routine of parents and patients right after birth. It is a very complex and heterogeneous disease and affects more than 40,000 people in Europe, with the number rising. The health condition of persons with CF can change rapidly. It can be positively influenced by good treatment, which renders daily, lifelong therapy essential. Many persons with CF tend not to adhere to parts of the therapy [11], making it necessary to develop an efficient way for self-managing the disease.

### The MyCyFAPP Vision

The research presented in this paper is part of a comprehensive user-centered development process performed in the European Union–funded research project MyCyFAPP [12]. The aim is to develop an app ecosystem (ie, an array of interconnected apps) that addresses persons directly affected by CF, parents of affected children, as well as health care professionals (HCPs) involved in the treatment. This app ecosystem is envisioned to bridge the gap between medical check-ups by providing the following:

- Tablet-based games for children to increase their knowledge about the disease and encourage to compliance with the daily therapy
- An app for teenagers to help them to take responsibility for the treatment
- An app for parents and adults with CF to organize the treatment and follow up the health status
- Support for health care professionals (HCPs) to follow up patients

The CF care experts involved in MyCyFAPP identified enzyme dosage as a key challenge for persons with CF. Most CF patients have to follow a pancreatic enzyme replacement therapy, where enzymes have to be taken with each meal to help digest food. The amount of pills to take depends on the type of food and in particular, on the interactions between fat and other nutrients in each meal. MyCyFAPP is also researching on the role of individual digestion conditions, but regarding this, no conclusion can be presented yet. Enzyme dosage is an essential part of the CF therapy, with practices on recommended dosage still varying across Europe [13]. A wrong dosage can lead to malnutrition and gastrointestinal problems [14]. In parallel with the design of digital support, the medical team in MyCyFAPP is currently developing an algorithm for dosage calculation. An aim is to integrate this algorithm in the digital self-management support.

The research in MyCyFAPP adopts a user-centered design approach following the European standard ISO 9241 in which potential users are involved in all stages of the development [15]. This is to ensure that the apps developed best suit the potential users. When developing an app for persons who are dependent on complex, precisely planned, and time-intensive daily treatment routines, this inclusion is crucial. In line with this approach, the following user-centered design steps are conducted in the project:

- Analysis of context and user requirements
- Iterative codesign workshops with all target groups for the creation of ideas
- Iterative user tests of first concepts and prototypes for the idea selection, realization, and evaluation of the solution
- Evaluation of the final prototypes in a 6-month clinical trial for a final evaluation of the solutions

This paper focuses on the first step of the user-centered design process, that is, the analysis of context and user requirements.

### Patient-Centered Care

Patient-centered care is a comprehensive approach to the patient-HCP relationship, which includes aspects of self-management, patient education, and clinical practice [16]. Self-management is particularly relevant for persons with CF. CF typically shows a high degree of individuality with great variability of the disease and related differences in treatment schedules and patient needs. Therefore, it may be argued that it is crucial for CF patients to be able to act as an active partner in their relationship with the HCPs.

A patient-centered approach to care will influence the relationship between the patient and the HCPs [17]. Irwin and Richardson [16] explain why both parties’ needs and perceptions are fundamental: they have to collaborate and share knowledge about the treatment and the course of the disease. This also implies that the patient accurately reports activities and symptoms and actively complies with the treatment routines by changing behavior in line with changing health needs. The HCP has a crucial role in determining individual treatment routines together with the patient. HCPs can support the patients’ self-management behavior between consultations, for example, by providing them with the necessary knowledge. Collaborative roles of patients and HCPs are discussed in more detail in other studies [10,18].

### Self-Management of Cystic Fibrosis

One way to foster patient self-management is through personalized apps. However, to deliver high-quality apps, one should base the development on thorough knowledge about user needs, which stems from a user participation methodology. To our knowledge, little research work has been conducted in the area of mobile digital self-management of CF. A search in the Scopus database for articles containing the key words “cystic fibrosis” and either the keyword “app” or “mobile application” returned only two relevant results in May 2015 [19,20]. Cummings and colleagues [19] describe a trial to evaluate the use of an app for CF patients to self-report symptoms and communication with mentors. Their research indicates that digital self-management for CF is promising. The trial demonstrates that the use of an app is feasible with a geographically dispersed CF population. The app was generally considered to be useful and allowed CF individuals to focus on changes in symptoms. However, the functionalities covered by the app were determined by the nature of the experiment rather than the users’ requirements. Building an app on top of user wishes, as it is being done in this research, is more likely to match their needs and converge into a highly usable and acceptable solution [21].

Hilliard et al [20] focus on user needs. Their study is built on questionnaires and semistructured interviews with adults (older than 18 years) with CF. Participants were asked about their preferences for an app for self-management. They found that persons with a smartphone would have the app to help them manage the disease. The study identified a list of preferred features for such an app. This list of features included, but was not limited to, access to health information, communication with other people with CF, communication with health providers, automation of the process to order medication, and tracking and visualizing health behavior. Hilliard et al [20] also describe some nonfunctional concerns such as the need to tailor the app to CF therapy in contrast to generic apps; the need of an app interface that requires little interaction from the user’s side (that does not take much of their scarce free time), the need of having a single app with multiple features instead of multiple apps, the possibility to customize app features, and privacy settings.

Hilliard and colleagues [20] present some initial expectations for a CF self-management app ecosystem that are highly relevant for our study. We go beyond the scope of both previously discussed studies as we include multiple stakeholders (parents, patients, and doctors), patients from different age groups (children, teenagers, and adults), and participants from multiple countries across Europe.

### Study of Existing Cystic Fibrosis–Related Apps

In addition to the literature search, searches on Google Play Store and Apple App Store (the two biggest app Stores) for CF-related apps revealed 35 apps that were available for free and could be used for self-management of CF. Apps used for fundraising for CF and one e-book app for a scientific journal about CF were excluded. All apps were downloaded and tried. The app search gave an overview of what is currently available to persons with CF. We found that the most frequent features are educational information, reminders for medications or events, medicine registration, symptoms registration, social networking, and guidance on how to perform treatment (specifically the chest massage). The few CF-related games available are about educating the patient; one about helping the child to perform respiratory therapy by blowing in the phone. The search inspired our interviews as it gave us ideas about features that could be included in an app for CF. It showed that some needs expressed in the study by Hilliard and colleagues [20] are not yet addressed by any app. In particular, there are no apps that facilitate communication with health providers, or that track and visualize health behavior. Looking back at the apps after the case study to be described in the paper, we see that none of them cover all the nonfunctional concerns raised by users. The apps often revolve around a single functionality, offer no customization, and often require time-consuming data input.

## Methods

### Research Strategy

**Figure 1:**
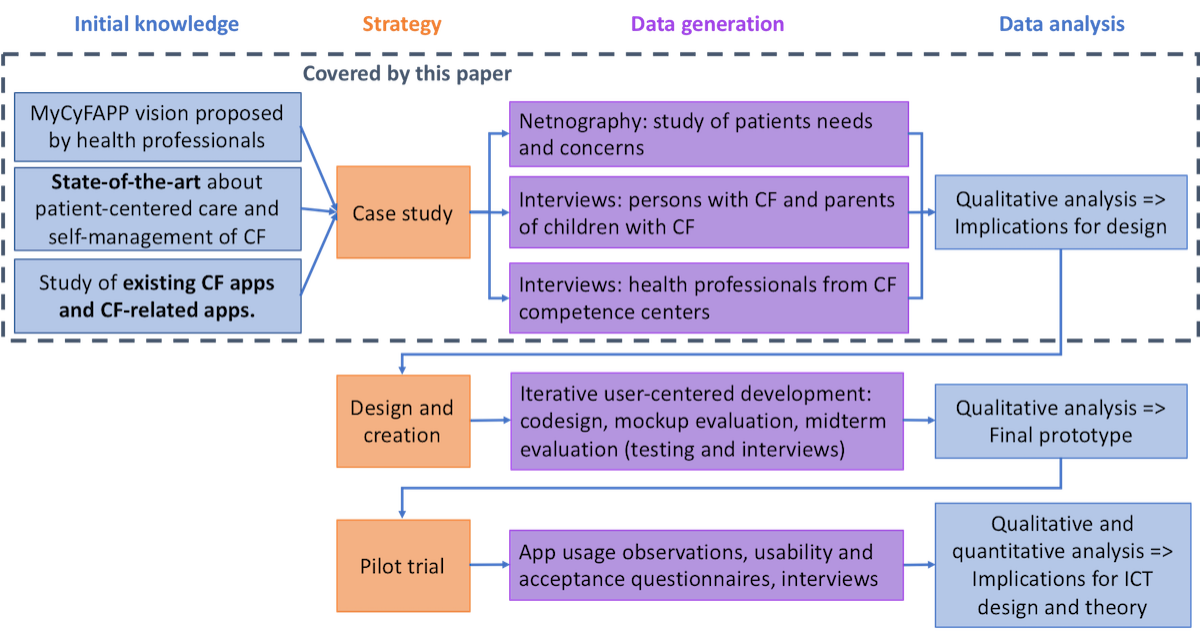
Strategies for the information and communication technology (ICT) research in MyCyFAPP. CF: cystic fibrosis.

Figure 1 provides an overview of the strategies for the ICT research conducted in MyCyFAPP using the research model process and terminology proposed by Oates [22]. Other research activities in the project, that is, medical research and food engineering research, are not included. Different research strategies are taken in use for the user-centered design steps:

- A case study is applied for the analysis of context and user requirements. A case study is an inquiry that focuses on one aspect to be investigated with the aim to obtain a rich detailed insight into the life of the case and its complex relationships and processes [22]. In our work, we study the context of care of persons with CF in depth. Related to the holistic framework proposed by van Gemert-Pijnen et al for the development of electronic health technology [23], this step maps to the “contextual inquiry” and “value specification” activities.
- Design and creation is applied for codesigning, developing, and testing ICT artefacts [22]. The artefacts are not solely software prototypes but also include paper prototypes and mock-ups. This step maps to the “design” activity in the framework of Gemert-Pijnen et al [23].
- Finally, a pilot trial will be conducted to evaluate the final software prototypes and provide evidence of the usefulness of the solutions. The trial is conducted by the CF care experts involved in MyCyFAPP as an experiment [22], investigating changes in physiological parameters and QoL following the introduction of the solutions. In the ICT research study, we do, however, not plan to compare the situations “before” and “after” the trial.

The case study, as well as design and creation, follow an interpretive research approach. We do not prove or disprove a hypothesis but rather aim at understanding the social context for the systems we develop.

This paper covers the case study, aiming to analyze the context and user requirement. We follow an interpretative case study research strategy [24,25] to answer the primary question, “What digital support has the potential to usefully support persons with CF and their caregivers in the CF care?”

This requires us to understand the current situation, and we therefore define the preliminary questions, “What important factors in everyday life affect the care of persons with CF?” and “How is the CF care delivered today and what are the limitations of CF care services?”

The context of the case study is the everyday life of persons with CF and their caregivers in seven European countries. We seek to shed light on the tasks needed for the CF care, the challenges encountered in the care, and to derive the implications for ICT support.

As a starting point for the study, we use different sources that were earlier summarized in the paper. First, the vision of MyCyFAPP was developed by experts in the CF care, working in six different CF competence centers in five European countries. In addition, a study of the state-of-the-art for digital self-management in health and the testing of available CF-related apps provided us with an initial understanding of the context. During the case study, data were collected through netnography and semistructured interviews.

### Netnography: Data Collection and Analysis

An online forum research based on the concept of netnography was first performed. This gave us a broader overview of the patients’ needs and concerns. Netnography is “a written account resulting from fieldwork studying the cultures and communities that emerge from on-line, computer mediated, or internet-based communications, where both the field work and the textual account are methodologically informed by the traditions and techniques of cultural anthropology” [26]. The method allows to collect questions and concerns from a broad audience among the CF community. It offers additional insights as the interaction is more anonymous than direct interviews. This potentially leads to more open discussion than during personal contact with a stranger. Five internet platforms (two in the English-language [27,28] and three in German [29-31]) were researched for entries about enzyme therapy, food intake, as well as details about patients’ daily life with CF.

All forums that were accessed were registration free, and the researchers did not participate actively in any discussion. The data collected were analyzed using the coding software Dedoose provided by SocioCultural Research Consultants, LLC (Manhattan Beach, California).

**Table 1:** Overview of the participants across countries and target groups.

Country	Target group						Total
	4-12 years	13-16 years	16+ years	Parents	Association	HCP	
Belgium	—	—	1	2	—	2	5
Germany	2	—	2	2	—	10	16
Italy	—	2	2	2	—	2	8
Netherlands	2	2	—	1	—	3	8
Norway	—	—	1	3	1	5	10
Portugal	1	2	2	2	—	3	10
Spain	1	1	3	5	1	6	17
Total	6	7	11	17	2	31	74

### Semistructured Interviews: Data Collection and Analysis

Qualitative semistructured interviews with persons with CF, caregivers, and HCPs with CF care expertise were performed face-to-face, as well as over the phone. Although persons with CF and caregivers describe personal concerns and needs, HCPs share general experience and experience-based advice. These viewpoints are complementary. All interviews were conducted individually, except for practical reasons that of three doctors and one nurse at the Norwegian CF center and of three doctors and dietitians in a Portuguese CF center. The study includes all relevant user groups: children and teenagers with CF in the age range of 4 to 16 years, young adults with CF older than 16 years, parents, and HCPs. HCPs include doctors, nutritionists, and nurses. In addition, members of CF associations were also interviewed, so that a total of 74 participants were included. They were recruited through national CF associations and hospitals in seven European countries. The participants could choose between face-to-face or phone interviews. Table 1 gives an overview of the participants across countries.

A central element of the study is to include several countries. Different countries organize their CF health services differently, have different digital routines, and cultural differences. The goal of including equal numbers per target group in all countries could not be achieved. Given that the treatment is time-consuming, accessibility to patients was a major challenge as it sometimes involved a lot of extra time and effort for the participants. Further challenges in recruitment were the fact that young children could not be interviewed over the phone leading to occasionally long travels to CF centers and that patients could not meet for group interviews because of cross-infection risks. In addition, children and teenagers who are supposed to be included in the planned clinical trial later in the project were not involved in the interviews to avoid a bias.

Slightly adapted interview guidelines were provided for children, parents, and HCPs. Individual interviews lasted between 30 and 60 min and the group interviews 3 hours. Topics included:

- Demographics, personal details, technology usage (all groups)
- General needs, fears, hassles, typical day in a life, motivation (persons with CF and parents)
- Self-management of the treatment vs support with treatment, motivational factors, obstacles or problems (persons with CF and parents)
- Experiences and expectations about food recording and enzyme dosage calculation (all)
- Communication between patients or parents and HCPs (all)
- Communication with other patients and parents (persons with CF and parents)
- Apps they like and/or use regularly (persons with CF)
- Expected features in a self-management app (all)

All participants were given information about the research and the management of collected data. They signed under a letter of consent. The interviews were recorded and transcripts of those written and notes taken for data collection and analysis. The transcripts were sent to interviewees for feedback.

The analysis of collected data was performed in an inductive way. The framework and the principles of Klein and Myers were used in this process [32]. The researchers read the written interview transcripts and performed a first independent round of thematic analysis [33] resulting in a number of initial topics. Then, a refined working set of topics was iteratively created through collaboration among the participating researchers.

## Results

### A Large Diversity of Needs

Over 450 needs were identified across the target groups. The participants in the study did not only describe their own experience and express their own needs. Parents explained difficulties encountered by their children. They have expectations about how children should tackle the disease, and they foresee useful app features for children. On the basis of experience from the past, parents with older children and adults with CF expressed needs on behalf of parents with younger children, children, and teens. Although the vision of MyCyFAPP includes a management tool for HCPs, HCPs found it difficult to express needs for a tool intended to support their own work. Their main concern was the digital support for the patients.

### Topics Identified Through Netnography

Results from the netnography research show that under a total of 32 main topics identified, enzyme is the most discussed one in user forums (51 mentions), followed by motivation and discipline (44), diet (38), weight (36), exercise (21), supplements (20), and time management (16). Topics can be further classified according to whether the user is a parent or a patient. The most important themes discussed by parents are difficulties with their children not eating enough, not gaining enough weight, and not taking their enzymes. Physical exercise appears to be more of interest for older patients. Issues such as time management and discipline are more often discussed by adults with CF. These results indicate the relevance of the topics suggested in the MyCyFAPP vision, in particular, support of enzyme dosage calculation and nutrition management.

### “What Important Factors in Everyday Life Affect the Care of Persons With Cystic Fibrosis?”

We identified the following main factors that influence the daily life of patients and parents of children with CF:

- CF is a serious disease and its treatment demanding.
- It takes time for caregivers and persons with CF to gain experience in the treatment and to establish routines necessary to handle it. The level of experience with the disease and the ability to set up routines lead to varying needs.
- Whenever there is a change in life circumstances, when the health condition changes, or when the patient grows older, daily life and the connected needs are affected.
- Patients behave differently, and the level of compliance to the treatment varies. They also accept the disease differently. Behaviors change over time too. Accordingly, the level of support to comply with the daily treatment and make life easier varies as well.

**Figure 2:**
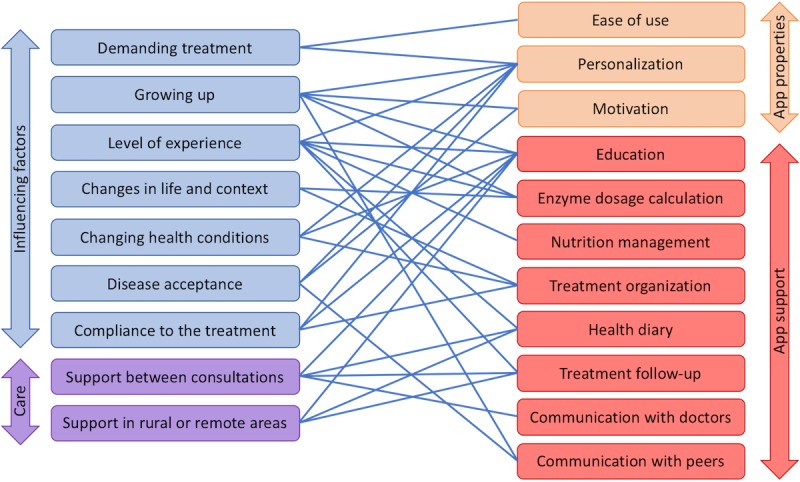
Overview of relevant app properties and features and relations to influencing factors.

In the following, we describe these main influencing factors and, for each factor, derive implications for the design of digital support. A summary of the implications and their relations to the identified factors is presented in Figure 2.

The interviews were conducted in different languages and most quotes included in this paper were translated by the authors. When English was used, participants were not English native speakers. Many participants in the study use Creon as medication for enzyme replacement, and therefore, referred to Creon instead of enzyme in the interviews. In the following, we use “parents or mother or father” and “child or teen or adult” for “parents or mother or father of child or teen with CF” and “child or teen or adult with CF,” respectively.

#### A Demanding Treatment

The treatment of CF is time-consuming. Every day, people with CF do some form of airway clearance, for example, chest physiotherapy, to clear the lungs from the thick, sticky mucus. This is associated with inhalations that help keeping the airways clean. Inhaled medicines may contain antibiotics to fight lung infections. A session takes between 20 to 40 min. Clearance may be needed more than once a day. The equipment used has to be kept clean to avoid infections. People with CF are also recommended to practice physical exercise regularly to improve their lung function, as well as heart and muscle function. One parent stated the following:

Yes, the day is busy. It starts early. First, 20 minutes medication and then getting everything else ready. And then there are times with infections, and he is in a bad shape and tired, and his motivation is low. We cannot say that it is not demanding. It is.

Mother of 8-year-old child

All users targeted by the apps in this study have a very tight daily schedule and limited time available, as illustrated in the following quote:

...usually one to one and a half hour[s] of treatment each day. The double if she has a serious infection… Lack of time is the most challenging problem

Mother of 7-year-old child

The experienced lack of time can make it difficult to keep motivated and to adhere to the treatment, especially to do airway clearance, the most time-consuming part of the treatment. A teenager stated:

It is hard to find the time to do physiotherapy.

17-year-old teenager

The lack of time conflicts with the wish to live as normal as possible, as illustrated in the following quote:

Time is the biggest problem. Most patients want a normal life. They have to go to school. Some adults want to work. They want to have a family. CF is not about just taking the pills in the morning and then everything is solved.

Doctor

It is important to set up routines and structure the day to both meet the needs of the treatment and give room to other activities. Two parents stated the following:

The nutritional aspects, the Creon, the medications, the physio, the aerosols, the homework...There is not much time during a day. I go to work in the morning while she is at school and then I dedicate all my time to her. Every day. However, we have automated this since she was born.

Mother of 10-year-old child

Follow-up of CF takes time and needs caution on a daily basis. It is paramount if the routines, for instance training, are integrated in the family life.

Mother of 8-year-old child

Implications for design: following the limited time available, ease of use and real added value are found to be of the highest importance for a future app. An app can hardly reduce the treatment load in daily life, but it can help enforcing routines. One parent stated the following:

The parents need tools to make the life easier and to have to be dependent as less as possible on the disease. To try to live as normal as possible.

Mother of 4-year-old child

It is not realistic to expect, neither should the HCPs require, that an app is being used every day. In the following, we will see that particular situations, for example, eating a new type of food or taking new medication, can make an app particularly useful.

#### Experience With the Disease

When the disease is first diagnosed, nowadays, usually a short time after birth, parents are eager to find out more information about the disease and its treatment, as illustrated in the following quote:

People with recent diagnosis often contact the organization before they visit the CF Competence Centre. They search on Google and get in contact with us often the same day as they are diagnosed.

Patient association representative

CF is a complex disease. It takes time to acquire understanding about the disease and its treatment. Parents with young children describe more problems with the treatment than other groups in our interviews. They need more support and contact their CF center more often, as illustrated in the following quotes:

Parents with new-borns need more communication.

Nurse

The patients contact the Centre when they need advice. Usually parents with young children contact us often.

Dietitian

It is important for persons with CF to have a good nutrition. Many persons with CF, as well as parents, fear that the diet is not healthy or rich enough. They worry that the enzyme dosage is not appropriate and might ultimately lead to nutritional imbalances:

Many of the telephone requests are about enzyme dosage. Many patients struggle with stomach pain [...]. Sometimes this is, because the patients are unable to calculate the correct amount of enzymes. Compliance to correct enzyme dosage is difficult in all age groups.

Patient association representative

Parents of newborns and young children face additional challenges, as illustrated in the following quotes:

Initially it was difficult to find out what was the right amount of Creon. Especially when he was a baby and he was still on mother milk [...]. It gets easier when the kids are able to eat by themselves.

Mother of 9-year-old child

The Creon before meals, it is a problem when the child doesn’t want to eat.

Parents of 3-year-old child

Even after more experience with the disease, parents and adults may find it difficult to calculate enzyme dosage, and some are uncertain about doing the right thing, as illustrated in the following quote:

It is a challenge to find out how much Creon to take.

19-year-old adult

However, the disease is individual. People with CF have different symptoms and challenges. Persons with CF and parents also learn to tackle the symptoms:

I have no problem with Creon. I am used to it. It also works well when I am not at home.

21-year-old adult

About enzyme dosage, parents said:

In the beginning, I had to search, but now I know it is 8 or 10 pills.

Mother of 15-year-old teenager

He gets 4-5 capsules a day, but I wonder if it is too little. I see on the internet that other children take much more at the same age. But he is growing, he is active and he has no constipation, so I assume he gets enough.

Mother of 2-year-old child

Maybe I am giving too much, but I do not think so, because he is doing well. There was a moment where he was not growing that much and was not taking weight very much. I tried to give more Creon and it seems that it was the problem because it was much better afterwards.

Mother of 17-year-old teenager

As parents and people with CF gain experience, they also establish routines to deal with the treatment. Although enzyme dosage was most often mentioned in relation to acquiring experience, routines relate to all parts of the treatment, as illustrated in the following quotes:

Her day is busy with exercising, medicine intake, school homework […]. We have to be well organized. We have routines that help to not forget anything, especially medication and physiotherapy.

Parents of 10-year-old child

Especially at the beginning when the routine was not in place, we had a paper sheet with a weekly table. In the rows, we had the different times for medication, what kind of medication. This sheet also serves today when he is going to his grandparents, when we organize the treatment and the therapy to be taken there. For us, no longer, because we have the daily routine.

Mother of 9-year-old child

Implications for design: the level of experience influences what digital support is needed. An app can help parents and patients to acquire knowledge about the disease and the treatment. It can also support them to develop experience in dealing with the disease and, particularly, to get more confident with enzyme dosage calculation. Furthermore, an app is well suited to set up and schedule activities and therefore, support the management of the disease. One participant stated the following:

Vitamins and antibiotics can sometimes be complex. It can be useful to set reminders for them. And to set the range of dates, because I may take a specific vitamin twice a month...

23-year-old adult

#### Growing Up

Growing up is another factor with a great impact on the life of children and teenagers with CF and their caregivers. Such as everyone else, children and teenagers with CF go through different phases as they grow up and mature. In different stages of their life they have different needs. Growing up, they take more and more responsibility and mature to young adults. An app has to take into account these different needs and levels of maturity and should support patients and parents at different stages of their development.

When children are still very young and first diagnosed, HCPs mostly speak to their caregivers about the disease; they teach them how to take care of their child and what to focus on. Very early support for parents and children is important, as ensuring the right nutrition at this early age is especially important for the further course of the disease, as illustrated in the following quote:

It is important for CF patients to have a good nutritional status. It is important that the dieticians can begin to give parents advices very early or directly to the children if they are old enough.

Dietitian

For young children with CF, most information from the HCPs is targeted to the parents. The children are often present in these conversations but may not be very interested in them, as illustrated in the following quote:

The parents get a lot of information, they learn about what they should do. The children are often no so interested. They play or do other things when the doctors explain.

Patient association representative

Therefore, young children mostly have only a general idea of their disease and treatment or even a false one: a father of a 5-year-old child explains to his child, “that he needs it [the treatment] because he ‘works’ in this way.” A mother of a 4-year-old child (on how she motivates her child to stick to the treatment): she tells her daughter that “she needs to take the pills and do the exercise because she is growing up.” When asked by her child, why her mother doesn’t have to take the pills, the mother answers, “I did, when I was a child.”

When the children become older, their knowledge about CF becomes a little bit more concrete, as illustrated in the following quote by a child when asked about what he knows about his disease:

That there is something not working perfectly with my lungs and that I have to do all the inhalation and take enzymes to feel better.

9-year-old child

This knowledge is very important, as when growing up, it is important for children and teenagers to start being able to deal with the treatment themselves. Children go to school where their parents can’t watch out for them, and teachers are not necessarily acquainted to the treatment of CF. One dietitian stated the following:

It is sometimes difficult to estimate the dosage of enzyme. It is easier with young children because the parents always observe the stool. When children get older, the parents don’t observe the stool all the time [...]. It is a bigger challenge for school-age children. Children have to learn when they are old enough about how much fat the food they like contains and how the stool should look like.

However, starting to take this responsibility for their treatment can be very difficult for some children and teenagers, as they are missing knowledge about their disease, as illustrated in the following quotes:

When they get to the point where they should take responsibility, the doctors forget that they have [only] taught the parents. They have the expectation that young people know, but may be one should start educating again.

Patient association representative

Facing adolescence it is important that she [the child] starts to become autonomous and to have that information without the mother being pending. So having a dual app will be important in the transition to adult ages. The idea is to “replace” the mother role and be more independent. For this, if there is an app to help it would be fantastic.

Mother of 10-year-old child

Therefore, one of the associations involved in the research exploited events for young people to educate them. For example, they organized quizzes and sessions to exchange experiences, as illustrated in the following quote:

Young people like games, so games might be used for learning. It’s more fun than listening to a doctor.

Patient association representative

Implications for design: an app can support HCPs and parents in teaching young children about CF in a way that is easy to understand and exciting. It can raise their interest and help avoid a knowledge gap for older children, allowing children and teenagers to slowly take over more responsibility for their treatment.

Starting to take responsibility for the treatment can be difficult for older children because they are missing information about CF, which earlier was mostly targeted to their parents. But also because puberty is a difficult time in general and especially difficult when having a severe chronic disease. HCPs and a young adult with CF stated the following about the difficulties of motivating teenagers to stick to the treatment:

Teenage time is the most difficult. They cannot participate to the same activities as their peers. Some think about suicide. At 20, one gets more mature.

Patient association representative

Teenagers often need more motivation. It is important to follow them up more. But this is for some teenagers, not all. Sometimes they find it difficult to follow the treatment because the transition from child to adult is not an easy period, as girls often want to be thin and boys want a lot of muscle mass.

Dietitian

When asked about problems for patients and parents regarding treatment, one nurse stated:

The rebellion phase of teenagers—it is very difficult. They want to know exactly why the drugs are important for them.

Nurse

One teenager stated:

When I was a teen, there were sometimes periods where I did not want to talk about CF.

19-year-old adult

As explained above, in general, parents take most of the responsibility for the treatment of their children before they go to school; starting school, children have to start taking responsibility and manage their disease more on their own; as teenagers, they have to become even more independent but often miss the motivation for it.

Despite this subdivision in very general age groups for the level of independent managing of the disease, there is much variation about how early children start taking responsibility. This depends on the children but also on their parents. Some parents give responsibility to their children very early. Other parents are very protective, and children are used to rely on them, even when they become young adults.

Some children and teenagers tell us that they started to take responsibility for their treatment (especially for their medicine intake) before or when starting school, as illustrated in the following quotes:

At 5 years old I took them [pills] by myself, at 7/8 years I did it all on my own...

15-year-old teenager

She knows everything since she was 4 years old. I give her the pills.

Mother of 8-year-old child

She got a lot of responsibility when starting at school, including taking medication on her own.

Mother of 7-year-old child

Other patients start taking responsibility only as teenagers, as illustrated in the following quote:

I started to be responsible for medication at 12 years old, for physiotherapy at 14 years old.

17-year-old teenager

There are different levels of responsibility. Some parts of the treatment are easier to adhere to than others. Often children start to take responsibility for their enzyme intake (Creon) first. For physiotherapy and other medications (eg, irregular medicine like antibiotics), as well as inhalation, they take over the responsibility later on, as these tasks seem to be more demanding, as illustrated in the following quote:

As a parent, you have responsibility. It is a question of maturity. We are not yet there. The Creon is much easier because he knows that before eating he has to take some pills, but we have to “stay behind [ie, supervise] for the aerosol and the physiotherapy. He won’t do that alone.

Mother of 9-year-old child

When asked about when they started self-management, two teenagers said:

Medication: 12 years old; Physiotherapy: 11 years old; Creon: 6 years old; I use the same methods as my mother to put them into boxes. My mum still reminds me for some new pills, but I’m independent.

17-year-old teenager

I started to manage Creon at 7 years old. My parents still remind me to take aerosols [inhalation] and Creon with snacks.

13-year-old teenager

But the more complex the diagnosis is, the more children and teenagers with CF have to rely on their parents, the more difficult it is to take over responsibility for the treatment, as illustrated in the following quote by mother of a child with very complex diagnosis:

She is totally dependent on me. She takes a lot of different medications that can cause negative interactions. I need something to manage this and be safer or to promote her independence once she grows; I control if she needs more Creon because she has diarrhea or less because she is constipated.

Mother of 10-year-old child

Furthermore, some parents find it difficult to give responsibility to their children as they want to protect them, as illustrated in the following quote:

We have to remind him. Maybe I am a too protective mother. I still prepare things for him. In the morning, everything is prepared, so he can start inhalation. Also when he comes to the table, the number of Creon is near his plate.

Mother of 15-year-old child

However, for most cases, the following rule applies: no matter the age and maturity level, most of the parents stay very involved in the treatment of their child, even when their children are already young adults. The role of the parents is to remind their children to not forget any step of their treatment and to do the treatment right, as illustrated in the following quotes:

[I stopped reminding him] when he was 8 years old, I still give him a paper, on which all treatments are written down (as a reminder), but he prepares everything on his own.

Mother of 9-year-old

Most annoying is the time lost, especially in the morning, it is exhausting to remember what to do when, mum sometimes helps with remembering and with instructions.

15-year-old teenager

At 7/8 years old he did both treatments and Creon intake autonomously. Nevertheless, we still remind him to take pills with him.

Father of 17-year-old teenager

I take responsibility myself. Sometimes my mother reminds me about the medication at lunch time. This works well. I started to take responsibility for the treatment when I was 16-17 years old.

21-year-old adult

Implications for design: with changing age, the purpose of the app changes. At first it is all about explaining CF and the treatment connected to it in an easy and entertaining and playful way, to ensure the attention of young children. Later on, it is necessary, to help children and teenagers to start managing their disease, at first together with their parents (eg, child registers food and follow-up by the parents), then on their own. Additionally, for all age groups, motivational elements play an important role: young children need to be motivated to learn about CF and to stick to the treatment and older children and teenagers have to be motivated to take responsibility for the management of their treatment. A digital support tool can help to ensure that children, teenagers, and young adults with CF can lead an independent life, and it can relieve parents from some of their responsibilities and therefore, make their lives a little bit easier (eg, by giving an overview of the treatment and offering reminders). An app has to be very flexible and adaptive to work for these different needs of users of different age groups.

#### Changes in Life and Context

Even though experience has been acquired and a well-functioning daily structure has been found, changes occur in life that affect the established routines. The first kinds of changes are those relating to life situations, for example, starting school or changing school, having the first job, or moving to another place, as illustrated in the following quotes:

She loves school, teachers and school mates. We were very nervous, teachers didn’t know anything about CF, we had to tell them all...It is very complicated...It worries me when she will go to the next school, we have to do the whole process again...

Mother of 10-year-old child

There have been some problems at school with bullying from the other children… There have also been problems with lack of understanding of the seriousness of the illness from some of the new teachers, because the child does not look ill in any way.

Mother of 7-year-old child

It went well when he was a child. [...] for 9 years he has been with the same friends. His friends know about him and they tell him to not forget his Creon. He changed school when he was 13. Then he was shy about it. He did not want to show that he was taking medicine.

Mother of 17-year-old teenager

It might be difficult to fulfil the diet and the pancreatic enzyme replacement therapy when they go to school or other activities out with friends.

Dietitian

Another kind of changes are short-term changes in the environment, such as eating out, going on holidays, or doing things out of the routine. Such changes were mentioned repeatedly as challenging situations, as illustrated in the following quotes, with the first quote on enzyme dosage:

When we go to restaurants, we don’t know how the meal is cooked.

17-year-old teenager

I have also an aerosol that I take three times a day, morning, midday and evening. The one at midday, I often forget because I am not always at home and I have no other aerosols at this time. At the university, I also lack time at lunch time to take the aerosol.

21-year-old adult

Furthermore, distractions can make people forget about their medication, as illustrated in the following quotes:

When many things happen, when things are joyful, then he forgets about it [Creon]. Afterwards he has so much pain that he knows this was wrong.

Mother of 9-year-old child

I know that I must take the Creon. Sometimes something happens that I have to take care of, and then I don’t know if I have taken it or not.

23-year-old adult

Implications for design: an app can help to tackle these different kinds of changes. Support for explaining other people, enzyme calculation, and reminders can be useful.

#### Changing Health Conditions

The health condition may change rapidly. For instance, the lungs of people with CF are prone to infection; treating these infections require antibiotics. For many, it is challenging to take a new medication, when the same medication has been taken for a long period, as illustrated in the following quote:

At a certain moment we had to go from 2 aerosol to 4 per day. [...] It was challenging to make it all fit before he is going to school. [...]. Mainly time is challenging, and if he has to get up earlier, motivation is also a challenge.

Mother of 15-year-old teenager

It is not always easy to learn about new medication. One patient association representative stated the following:

There are many things to learn when you get new medication. People get too little information about what the side effects are and how to administer the drug.

The health condition can have an effect on the ability to adhere to the treatment, as illustrated in the following quote:

When I am sick, I lose appetite and I lose weight.

21-year-old adult

The severity of the disease increases over time, and related diseases such as diabetes may occur, as illustrated in the following quote:

Now the treatments are harder than when I was younger. I had less things to do before...Physiotherapy is the most stressful.

17-year-old teenager in severe condition

The following quote is about diabetes:

It increases the complexity of managing the nutritional aspects. From the CF viewpoint, I need to take more fats and eat more and, from the diabetes viewpoint, I need to reduce sugars and fats.

23-year-old adult diabetic

The interviewed parents and persons with CF did not share their worries about health deterioration with us, but HCPs describe health deterioration as an important concern, as illustrated in the following quotes:

Nutritional problems increase with the disease severity, and often when the lung disease escalates...Creon and nutrition often stabilize when patients grow up before lung diseases appear again and ruin it.

Doctor

Some have a disease such that whenever they are at the consultation, we identify a new complication or a new disease, and a new negative message enters the line of others. This is hard.

Nurse

They fear that they get worse or their nutritional status deteriorates.

Dietitian

Implications for design: it is not realistic to expect that an app can solve all challenges risen by the severity of the disease, but helping to maintain the health condition as good as possible is a leading motivating factor for an app in the first place. An app can support tackling new medication and can help learning about new diseases. An app can also motivate to adhere to the treatment.

#### Disease Acceptance and Openness

Another important factor affecting the life of children and teenagers with CF and their caregivers seems to be how open they deal with the disease, as well as their acceptance of the disease and all that comes with it.

This level of openness as well as the acceptance differs quite a lot between patients: some children as well as teenagers with CF try to hide their disease, they don’t want others to know about their disease, or only tell close friends about it, as illustrated in the following quotes:

Some are ashamed of their disease, they try to hide it in school. They use a bag for sweets to hide the medicine.

Nurse

Youngsters [...] don’t want to do the therapies/enzymes in school, they don’t want to show that they are sick.

Doctor

A mother of an 8-year-old child sees CF connected with a public stigmatization and tries to protect her child from this by making him look like an “ordinary schoolboy,” as illustrated in the following quote:

We have taken over the responsibility for physiotherapy...He is an ordinary schoolboy with physiotherapy in the evenings. Thus he is released from the stigma attached to CF at school.

On the other hand, some patients speak about the great support they are receiving, especially from their friends because of being open about their disease, as illustrated in the following quote:

When I was little, it was a little difficult, the people in my class helped me. I have lots of friends, they all help and motivate me...

15-year-old teenager

Especially, friends seem to have a great positive impact on the motivation of young persons with CF and can help them to stick to their treatment.

In the experience of patient association representative, dealing with the disease is easier for those who speak openly about it, as illustrated in the following quote:

Some people are open about their disease while others never tell anyone. Those that are open often handle things better. Some are afraid that they would not get any job, if they tell.

Not only do some children and teenagers try to hide their disease, some also find it very difficult to accept the disease or having to do treatment because of the disease, as illustrated in the following quote:

It is difficult for her to understand or accept the illness—in her eyes she does not need the medication. She gets pain in her stomach if she does not take Creon, but even so she forgets or ignores to take Creon when she gets something to eat from others, for example an ice cream at a friend’s house.

Mother of 7-year-old child

Accepting the disease or the treatment seems to be especially difficult for younger patients, as illustrated in the following quote:

My mum sometimes tells me that I have a better life than many kids—but that is not true, they don’t have to do all the treatments. [...] My family wants me to go to Amrum for rehab, but I don’t want to! It will be like being in a hospital for weeks! My grandma told me she will get an Ipad for me when I go, but I don’t want to!

9-year-old child

Other children too don’t want to do the treatment or they don’t do it properly, as illustrated in the following quote:

They watch TV, cartoons, while doing the aerosols. For physiotherapy is different, the child needs to be concentrated...Sometimes, it is difficult because he just wants to play.

Parents of 5-year-old child

To help parents and young children with a lack of acceptance at this age, a nurse explains to the children in an easy way the benefits of sticking to the treatment. Especially important seems to be to explain to them that with sticking to the treatment, they will feel better, as illustrated in the following quote:

They [young patients] need to know that they could live well, if they take the medicine. I often ask them: “what do the enzymes in your body?” At the age of 5-6 I start teaching them about enzymes. [...] I help them to get around obstacles. I tell them that many scientist are working on the disease to find better treatments, I show them adults. I help parents to meet each other. I help them to motivate to take the medicine so they are better. I tell them: “you’re growing, congratulations!”

Nurse

Other than the younger patients, most teenagers seem to have understood that without compliance their well-being or even their life is at risk. They seem to have accepted the extensive treatment and the limitations caused by the disease pragmatically. When asked about problems with the treatment they stated the following:

I have to do it [the treatment], because I have a disease.

15-year-old teenager

I know that my wellness depends on it and I have internalized the processes. I don’t think too much, I do it.

17-year-old teenager

Overall, openness about the disease and acceptance of the disease seem to be very important for the compliance to the treatment. Openness about the disease makes support from others possible. Acceptance has a great impact on the compliance to the treatment, and the acceptance of the necessity of the treatment seems closely connected to the realization that only compliance to the treatment can ensure feeling well and staying alive.

Implications on digital support: to foster openness and acceptance, especially young patients need support in form of explanation of the disease and motivation to stick to the treatment. Easy to understand knowledge about the disease and treatment should be made available. Additionally, prejudice must be reduced to avoid stigmatization, and patients need support to be able to easily educate other people about their disease, so social support becomes possible, which seems very important for increasing and holding up the motivation of patients.

#### Compliance to the Treatment

Persons with CF are more or less concerned about following the treatment and food recommendations. Some strictly adhere to the treatment, as illustrated by this quote:

I do not have to do anything to motivate him to comply with the treatment. He knows that he has to do it if he wants to be healthy. It is not the right word. To be as good as he is now.

Mother of 15-year-old teenager

One teenager stated the following about the daily treatment:

I have to do it, so I do it...because without I don’t survive...

15-year-old teenager

There are different reasons for not being compliant. It is important to be well-structured. Two participants stated the following:

It’s sometimes difficult to remember if I have taken the medication or not. Maybe I am doing something else or something arises, and then I don’t know [...] I have a [...] brother with CF. He is not responsible at all, so I take care of him.

23-year-old adult

Some physiotherapy and medication can be missed because she forgets.

Parents of 10-year-old child

Sometimes persons with CF downplay some aspects of the treatment to “live a normal life,” as illustrated in the following quote:

Youngsters, they don’t eat enough, they don’t eat the right food. They don’t want to do the therapies and take enzymes at school. They don’t want to show that they are sick.

Doctor

Different parts of the treatment are difficult to adhere to, as illustrated in the following quotes:

It is not easy to take enzymes. And, in all age groups, compliance with Creon intake is difficult.

Doctor

Physiotherapy is very important. [...] The patients really do not like to do it. It is time consuming. Coughing out sputa is also annoying.

Doctor

As earlier explained, age also plays a role for compliance. Both physiotherapy and nutrition were often mentioned as challenging for young children. Parents said the following regarding physiotherapy:

Physiotherapy works only if the child collaborates.

Parents of 3-year-old child

Regarding nutrition, some parents and a dietician stated:

The little one doesn’t want to eat at the kindergarten, because he doesn’t feel like home there.

Mother of 4-year-old child

Especially in the kindergarten, he is a very bad eater. He doesn’t like a lot of things.

Mother of 3-year-old child

Sometimes children have eating problems. An app that motivates them to eat is useful for those patients.

Dietitian

And as also explained earlier, teenage time is a difficult period. Some teenagers wish to focus on other aspects of life than on health issues and daily treatment, as illustrated in the following quotes:

Regularity and discipline are important. [...] It is tough to motivate teenagers.

Doctor

Girls from 10/12 years on don't want to gain weight.

Doctor

Doctors and parents also explained that children do not always understand the need of lung therapy, which has a long time effect. It is therefore important to motivate to adherence to that part of the treatment, as illustrated in the following quotes:

Physiotherapy and inhalation are challenging. The effects of not adhering are not visible at once as for Creon. Young people do not think about that life may be some years shorter.

Patient association representative

Patients don’t always feel a negative influence if they don’t follow their physiotherapy or do not do inhalation. This part of the treatment is important for their future, but it is challenging to spend more than 2 hours a day for long time effect. Most of the patients take their Creon because they get stomach pain when they don’t. Taking pills does not take time either. Inhaled drugs require time.

Doctor

Parents use different approaches to motivate their children, as illustrated in the following quotes:

We run together everyday. We often run past a shop and buy ice cream or candy to motivate him. We have trails of different lengths. I try to make it fun...It is important that training is not something he must do alone. He must do this every day of his whole life, and therefore it’s important to do something nice. At the same time, he is “allowed” to have some bad days. Then we walk instead of running.

Mother of 8-year-old child

If things are done properly, we take the bicycle, we go for a quick walk, we go to eat an ice cream. Things like that. Small things. I do not want to promise things totally out of scope.

Mother of 9-year-old child

Medication was like a game played with my mother.

23-year-old adult

It can be hard to motivate over a long time, as illustrated in the following quote:

It is sometimes difficult to motivate. It feels unfair to be different...We have used stickers as awards, and we had agreed upon some items after collecting a certain number of awards, but it loses appeal after a while.

Mother of 8-year-old child

Most of the children in our study receive good support within their family. But the level of support may also vary, as illustrated in the following quotes:

We had family problems and were not motivated to learn [...] I started very early in my life to take care of my young brother.

23-year-old adult

We have regular consultation with the dietician in the context of the quarterly control. We receive advice to improve his appetite [...]. We follow as much as we can.

Mother of 9-year-old child

Implications for design: support for learning about the disease and for understanding the purpose of the treatment and how it works are important for compliance. Reminders and checklists are simple means for those who forget. Additionally, support for motivating, for example, through gamification or playful interfaces, should be considered.

People are, however, more or less rigorous. It is not realistic to expect that an app will solve the problem of compliance. One HCP said the following with respect to considering a diary as a means for learning and reporting to doctors:

Who will reveal they are not doing what you have told them? Registering information is demanding and revealing. People are very different. Some do not have any overview and others have neat handwritten notes about everything that has happened day by day.

### “How Is the CF Care Delivered Today and What Are the Limitations of Cystic Fibrosis Care Services?”

CF is a complex disease whose care requires a multidisciplinary team approach. All countries involved in the study have established CF centers with specialized expertise [34]. The patients meet regularly at the center for consultation, usually every 3 months, and even more depending on the severity of the disease. A HCP, an adult with CF, and a parent stated the following:

The centre has a multidisciplinary clinic where patients come and are seen individually by pediatrics, nurses, physiotherapists, sometimes by a psychologist and sometimes by a dietician. The patients come every 3 months for consultation. Some caregivers, the physiotherapists and dietician do not see the patients every time they come. They try to see every patient at least once in a year. If they have problems or special questions, they see them more often.

Doctor

Normally I go to the doctors every three months, except if things do not go well.

21-year-old adult

We are going every three months to the control. It is a multidisciplinary team. When you go, it is often half a day, one afternoon for example. You see the dietician, the physician and the lung doctor. All these professions because they cover the disease together...Between the controls, they are always reachable by phone.

Mother of 9-year-old child

A more thorough control is often done every year and might require collection of food records and a stay at the hospital. One HCP stated the following:

Depending on needs, the yearly control lasts from one to three days. For some it may be a bit longer.

Doctor

Besides regular consultations, patients also contact their CF centers via other communication channels. Nurses are the first contact point for the patients, as illustrated in the following quotes:

Patients can communicate with the CF centre between consultations every 3 months. The centre has a central phone number and email address. The nurses and secretaries take care of messages. The nurses may answer themselves. If they think a doctor has to answer, they forward to one of the doctors. If every patient contacts a specific doctor, the centre loses overview of what is happening. Using phone and email, the patients avoid coming to the centre when they have a small problem.

Doctor

We have continuous contact with most patients by phone and letter. We do phone consultations. [...] We are now establishing a new video communication service. [...] We have contact with the vast majority of patients every year. The threshold to contact us is low. The critical issues are handled face-to-face.

Nurse

I do not usually contact the doctors between consultations, except if I get really bad...Normally I send an email and the nurses handle the request. I never contact the doctors directly. This works well. I get answers rapidly.

21-year-old adult

Allocating staff for phone communication might be challenging, as illustrated in the following quote:

All patients say that telephone consultations are annoying during the daytime. Everyone is at work during the daytime. Phone works in the afternoon and evening.

Nurse

Sometimes information is not properly registered, as illustrated in the following quote:

The information is not always correctly registered. For instance, when a patient contacts the centre to tell about a problem with a drug. Somebody may tell the patient to change to another drug, but not record it in the system. So when and why the change is done is not recorded.

Doctor

Besides health follow-up, the CF centers also teach and provide recommendations to the patients, as illustrated in the following quotes:

Inhalation is challenging. This is a big responsibility for the nurse and for the physiotherapist. They try to instruct the patient in the correct way and to evaluate. When the patients start a new drug, they try it for the first time during the consultation to see if they can do take it properly.

Doctor

I communicate with patients also by phone and email, if they have questions or problems. I give advice for the enzyme supplement. I look at the weight evolution, at the height evolution, and adapt the enzyme supplement in order to increase weight and height.

Dietitian

Some CF centers also provide external support, as illustrated in the following quote:

We travel to schools and kindergarten and provide recommendations. Local physiotherapists may also ask for assistance.

Doctor

An important part of the care is the daily physiotherapy. Although some parents learn to do the physiotherapy themselves, some prefer to rely on physiotherapists. Physiotherapists may do home visits. However, not depending on physiotherapists is also important with respect to independence. A HCP and two parents stated the following:

Patients can learn to do physiotherapy themselves. This is what most patients do. Patients know what they have to do, to clear their secretion. However once or twice a week, they go to the physiotherapist. The physiotherapist can apply more pressure on the thorax and feel, where secretions are, so it is better.

Doctor

As far as the treatment is concerned, he is very conscious and does it in a responsible way. He goes to physiotherapy every day, 7 days a week, even Saturday and Sunday. [...] We have learnt ourselves too, but do not do it as good as professionals. We know just enough to be able to go on holidays...Now he is older, so if we are doing something wrong, he can tell how to do it.

Mother of 15-year-old teenager

Physiotherapy depends on his status of coughs. It can be one, two or more times a day. [...] Fortunately, the physiotherapist is coming to our home. On the weekends and if we go on vacation, we give the physiotherapy ourselves. It is preferable that a professional gives the physiotherapy.

Mother of 9-year-old child

A good nutritional balance is extremely important for CF patients. It is, however, challenging to keep track of the nutritional status. The CF competence centers involved in the study sometimes ask their patients to perform a 4-day collection of detailed food records. Recording can be done in connection to the yearly in-depth consultation, or requested by HCPs when a particular problem occurs. Regular nutritional protocols can help centers to keep an overview of the nutritional intake of the patients and to identify possible reasons for occurring symptoms. But neither the patients and caregivers nor the HCPs are pleased with the procedure and the results of it.

For patients and caregivers, the tracking of the food intake can be very inconvenient, time-consuming, and difficult, as illustrated in the following quotes on difficulties with filling in the nutritional protocol:

Recording the quantities. You forget any ingredient and then it must be rechecked.

17-year-old teenager

The weight of each ingredient. You should introduce it in grams but it would be easier to introduce “standard” measures (a small spoon, a glass, a cup of coffee...). Additionally, there are many different ways to cook the same plate. It should be personalized as you cook and the ingredients that you use for doing a plate and how to cook them.

Mother of 10-year-old child

For HCPs, precise information within the protocol is very important; often they would need more detailed, accurate, and complete information in the protocols:

It [the nutritional protocol] must be as precise as possible regarding ingredients, amounts and cooking process.

Dietitian

One HCP stated the following about typical difficulties with the nutritional protocol:

Not enough details, incomplete information and too vague amounts.

Doctor

Although overall the CF centers function well, and patients expressed satisfaction in the care services they get, the availability of services is not always good for those living in rural areas, or those followed up by overloaded CF centers in large cities, as illustrated in the following quotes:

Some patients live quite far away, it is very expensive for them to come to the consultations and check-ups. It is very expensive. And transit doesn’t get paid.

Doctor

In comparison, at our centre it is good, 4-5 doctors for 120 patients, this is good. In Munich [big city in Germany] for example, 4-5 doctors are responsible for 400 patients. The amount of patients is increasing, the relevance is increasing. There are rural areas, where the care key doctor/patient is worse.

Doctor

In Norway, the care is organized differently than in the other countries involved in the research. The country is large with few people with CF spread all around the country. The policy is to give care to inhabitants close to the place they live. People with CF have their regular follow-up at a local hospital and a yearly consultation at the CF center (except those living in the Health Region South-East who are followed up by the CF center outpatient clinic). This organization of the care service received much critic both from HCPs and patients, as illustrated in the following quotes:

It is a challenge to establish good medical and interdisciplinary disciplines in Norway, which is necessary to deal with a condition as complex as CF. [...] It is important to understand that professionals in local hospitals, who may have one to three patients, do not have the opportunity to accumulate sufficient experience.

Doctor in Norway

Today, we have the main follow-up at the CF centre once a year. In between the follow-up is done at the local hospital once a month...I am concerned with nutrition and do not always agree with the local nutritionists, those who are not CF specialists...It is difficult because the doctors at the competence centre sometimes say something else than the doctors at the local hospital, for instance about taking antibiotics or not...

Mother in Norway

Beyond the policy encouraging local care, the transition to adult care also rises challenges, as illustrated in the following quote:

In ages from 15 to 25 [...] Moving from pediatrics care to adult care can also be challenging. Children often get more support than young people.

Patient association representative

As physiotherapy is concerned, people in Norway cannot count on a service available 7 days a week. Such does not exist. Parents are encouraged to take responsibility. One parent stated the following:

I give the daily physiotherapy following instructions from the local physiotherapist. The local physiotherapist was trained by the CF competence centre. The local physiotherapist comes home once a week.

Mother of 2-year-old child

Implications for design: the CF service care functions mostly well. In the case of rural areas or large cities, where the availability of the services or the expertise might be enhanced, we observe that patients are eager to take responsibility and manage CF by themselves. This indicates a readiness for the adoption of digital support for self-management. Additionally, in the case of a well-functioning care service and tight follow-up, we understand that there is a potential for letting patients manage more by themselves and understand more. Furthermore, some processes such as recording food could be simplified for all involved parties by supplementing or replacing them with a digital solution. Furthermore, we understand that there is a need for support between consultations, as illustrated in the following quotes:

Sometimes when I ask a mother more details about symptoms, for example “when does it happen?” she cannot always answer, and I see that she feels bad about not remembering. It is difficult for me too. I know that she spends a lot of time caring for her child. It would be helpful with an app that helps remembering what happened.

Doctor

When you go to the doctor, you tell about how you experience things, and you may tell in different ways. What cannot be measured objectively is your own experience. An app can help the patients and parents to structure their experience and to reflect about what happened. People may have the same disease, but experience it in different ways.

Patient association representative

### “What Digital Support Has the Potential to Usefully Support Persons With Cystic Fibrosis and Their Caregivers in the Cystic Fibrosis Care?”

On the basis of the data gathered as described above, we derived a list of main properties and support functions our CF app (or apps) should have to support patients in an optimal way. These properties and functions will be explained in more detail in the following section. Figure 2 gives a summary of this list and shows how app characteristics correspond to the identified needs of the users.

#### Ease of Use

Keeping in mind the very *demanding treatment* and very busy schedules of children and teenagers with CF and their families, providing support through an app makes it important to develop an app that is not “just another thing to do.” The app has to provide an added value to existing support structures and routines, make life easier, and save time. Therefore, the ease of use of digital support tools is exceptionally important, as illustrated in the following quote:

The app should be easy to use! Easiness and quality should be pointed out!

Doctor

On being asked if they would be willing to use a CF self-management app, parents stated:

I would register yes. It must be simple and easy. No writing, just select options after one first configuration.

Parents of 3-year-old child

#### Personalization

Not only is the ease of use of apps for children and teenagers with CF and their families essential, such apps also have to be customizable. The need for such *personalization* is stemming from the variability of the disease and the individuality of the patients. Despite having the disease in common, the patients do not necessarily share the same treatment routines, knowledge about CF, and other personal characteristics such as hobbies and life circumstances.

The *level of experience* of patients and relatives varies quite a lot: some patients and parents need more knowledge, for example, especially when they are newly diagnosed. Others, who are not confident yet with the dosage of enzymes, need more help with this, whereas others only need support, calculating the right amount for special occasions. Digital support has to be adaptable for these different needs.

Furthermore, when *growing up*, the purpose of the app changes: at first, gaining knowledge about CF is most important; teaching children in an entertaining way has high priority. Older children are supposed to start managing CF on their own. Motivating elements are needed at all times, but they have to be adaptable to the needs of the different age groups as well.

Furthermore, depending on the patients’ *health condition*, diverse functionalities within an app are needed. Patients with a deteriorated condition need different support to manager their disease. One parent stated the following when asked about the necessity of an alert or alarm:

Not for Creon, it is usual, [...] (And asked about the necessity of reminders for other treatments): Not, if they are as now. But if they would increase, it could be useful.

Father of 5-year-old child

When asked about the need for reminders for other treatments, another parent said:

It could be useful for antibiotics because it is distracting the routine of the daily treatment. It would not be a bad idea to have a reminder. It is true that sometimes the antibiotics treatment can be hard to follow.

Mother of 8-year-old child

With differences in the level *of acceptance of the disease,* needs may differ in terms of how much children and teenager with CF would like to communicate with peers or other HCPs. On the other hand, for persons with CF who are having trouble accepting their disease, motivational elements are immensely important, and education about CF within the app could be more helpful than for others.

Finally, the *access to specialized medical CF care* varies between countries, as does the *availability of education* about CF. In the case of Norway, for example, there is only one CF competence center, as compared with a large number of specialist CF care centers across Germany. The way the specialist CF health services are organized can hence influence access to expert consultation and *support* that some patients would ideally need.

#### Motivation

Parents and doctors express a need to increase awareness of possible negative consequences of noncompliance with the treatment. This is strongly linked to a need to get *motivational support.* Low motivation may lead to decreased treatment compliance. To bolster motivation should hence be explicitly targeted by the app. This is a challenging task, as illustrated in the following quote:

[Motivation] is difficult because it [the treatment and the disease] is individual for each patient. You cannot tell a patient that if you do your physiotherapy, your lung function will improve by 5%. We cannot say that because it is like a promise which may not come true.

Doctor

Participants stressed that a focus on the positive aspects of compliance with treatments and nutritional recommendations will increase motivation more than information on risks connected to noncompliance, as illustrated in the following quotes when asked about ideas for the app(s):

Please very positive messages! Supportive!

Nurse

You can better motivate with height [...] A game is helpful, how the disease works, understanding what doctors are saying [...] a quiz is always good! Rewards also!

Doctor

#### Education

There is a need to explain the disease and the importance and purpose of the treatment to young children in an entertaining and motivating manner, to ensure their interest and compliance to the treatment, and to avoid that they are missing important knowledge about their disease when they are already supposed to start taking responsibility for their treatment. This also can improve the compliance to the treatment in early years and can help parents to speak about CF with their children. Later on, there is also a need to gradually teach older children and teenagers how to take responsibility with respect to their treatments, as illustrated in the following quotes:

It is important that the app gives information directly to the child so they get motivated to eat well and take their Creon well. It is important that the children get motivated and that they get knowledge about their food and Creon.

Dietitian

For the children, it is important to educate them by entertainment. It is important that children know what they are doing and why. Often parents do all what they have to do, but 9-10 years old children cannot tell why they are doing it. Children should be instructed from the beginning.

Doctor

A Game would be helpful. How the disease works, understanding what the doctor is telling. [...] A Quiz is always good, rewards also!

Doctor

As elaborated above, especially parents of newly diagnosed children need support in learning about this disease, which is complex and difficult to understand. Educational elements in an app can help them to learn about the disease and treatment and to gain experience in dealing with it faster and therefore to help reduce some of the concerns of the parents.

Furthermore, educational elements in an app can be used to explain CF more easily to others. This is important when deviating from daily routine (eg, eating out) and especially at times of *changes in the life* of children and teenagers with CF (eg, changing school). It can also help children and teenagers with CF, as well as their caregivers, to be more open about the disease and to avoid stigmatization, as illustrated in the following quotes:

She does not like to talk about the disease. It could be easier if she had an app and could give her phone to an adult when she is visiting them, and tell them “look, this is how it works”.

Mother of 7-year-old child

He has to take his medication at school. We (I and his father) met the teachers and the director of the school to present CF and the implications in terms of treatment. They are following up for the intake of Creon. But you have to train them first to make sure they are following up.

Mother of 9-year-old child

Some are afraid that they would not get any job, if they tell. It could be useful with quiz etc. for those that are around them.

Patient association representative

Furthermore, an app can support patients and parents in learning about new medication or possible new diseases caused by CF and therefore, help them to better handle and to more easily adapt to a *change in their health status*, as illustrated in the following quotes:

Information for medicine usage would have been useful. There are a lot of questions about it in the Facebook groups.

Mother of 7-year-old child

It would be useful if doctors/dieticians can explain possible side effects related to food or medicines.

16-year-old teenager

Some patients seem to struggle with the practical aspects of the treatment, such as doing inhalation, taking care of the equipment, or performing physiotherapy. An app may provide information about practical aspects of the treatment in an instructive way. A parent stated the following about inhalation:

It would be nice to have some app that takes the time. Something that also shows him how to do it correctly. He has to close his mouth, to put it right in his mouth. Also, the breathing he does—it would be nice if there was something that shows him and takes the time.

Mother of 17-year-old

One patient association representative stated:

There are many things to learn when you get new medication. People get too little information about what the side effects are and how to administer the drug.

#### Enzyme Dosage Calculation

Enzyme management is especially important when routines are not yet in place or when patients and parents find themselves in *new situations they are not used to*, for example, when eating at a restaurant, and also when children *start taking responsibility* for their enzyme intake. But even teenagers and young adults who have been taking enzymes for many years are sometimes still *uncertain about whether they take the right dosage* or not. Supporting persons with CF and their caregivers with the help of an enzyme dosage calculation feature can improve the compliance to the treatment and foster their confidence with the calculation of the enzyme dosage. Some participants stated their wishes for a CF self-management app:

An app where one can take pictures of food, enter information about height and weight, how much Creon is taken, and then provide information about how much Creon is actually needed.

Mother of 8-year-old child

I would consult it [app] if I think the actual Creon dose is not enough for what I’m going to eat...and the app recalculates the Creon dose.

17-year-old teenager

An app that calculates the amount of Creon for various types of food would be useful.

20-year-old young adult

Enzyme management provides a personalized proposed dosage of enzymes depending on the composition of each meal and an individual correction factor of the patient. *Food recording* is thus a necessary feature for the calculation of enzyme dosage. For this food recording, the same level of detail is required as for the nutritional protocols mentioned above. Also mentioned above, this food recording has to be as easy, fast, and effortless as possible to avoid burdening patients and parents with this and to ensure added value by using the app(s). Cumbersome usage can be a reason to not use the app(s) at all, as illustrated in the following quote:

A calculator for Creon would be nice because sometimes it can be difficult to know what caused stomach pain. This may be due to too little Creon, or caused by other things...I am not sure how much time I want to spend to register food to get recommendation about Creon.

19-year-old adult

#### Nutrition Management

A good nutrition is essential for maintaining good health. Persons with CF have to follow a high caloric diet. A high amount of fat is the easiest path to achieve it, but it is not optimal for the health. One HCP stated the following:

Patients have a high caloric diet. It is a challenge for the health. It is important that they have a varied diet. People should not only eat biscuits, chips, ice cream. They should eat fruit and vegetables too.

Dietitian

Parents wish to compose meals that are both nutritious and healthy. Sometimes children get bored with eating. Dietitians provide advice during consultation, and this is something teenagers, adults, and parents would like to retrieve through an app. Two adults with CF stated the following:

It would be good with food recipes and advices. Often, I get sweet recipes, but I prefer salty food.

21-year-old adult

It would have been useful to get an overview of what is good to eat. I have lacked this. It is important to get good eating habits.

19-year-old adult

Furthermore, parents and adults wish variation in their food. They would like to be able to share recipes with other people in the same situation, as illustrated in the following quote:

It would be useful to exchange recipes and ideas about food as a source of inspiration.

Mother of 7-year-old child

Some parents, especially when not experienced with the disease, are keen about a detailed follow-up. They spend time calculating the nutritional composition of meals, especially fat, when their children are young. One parent stated the following:

When he was little, we used to calculate nutrition, especially fat, in all ingredients. [...] As for now, the calculations are routine based.

Mother of 7-year-old child

An app can support such calculation. Additionally, an app can also help to enable the dietitian to set up personalized nutrition goals in terms of energy and percentages of fat, proteins, and carbohydrates and to follow up the progress with respect to goals.

As already mentioned, today, patients are sometimes asked to provide detailed food records, but no tools are provided to support recording, as illustrated in the following quote:

We newly made a food record for four days when the dietician asked for it, and then one day a week later. The dietician provided a list but it was rather complicated because we had to fill it manually. Finally, I gave her an Excel sheet with meals and details about the food.

Mother of 7-year-old child

Food records are not useful if not precise. Dietitians think it is not realistic to expect detailed recording over a long time. An app should simplify recording for usage over long time. Two HCPs stated the following about food records:

Normally, they have to be as specific as possible. I ask for ingredients and make a caloric calculation. When I ask for a longer period, the calculation is not so good.

Dietitian

The app should support food recording, but asking to record for more than 3-5 days is difficult. The motivation is getting lower over time.

Dietitian

As an alternative to detailed food records, some would like an approximate recording as support for a consultation with the dietitian, as illustrated in the following quote:

It would be useful with support to note approximately what he has eaten during a day in order to give information to the nutritionist.

Mother of 7-year-old child

#### Treatment Organization

Routines are important with respect to making life easier and for the compliance to the treatment. An app can support the management of the different tasks related to the treatment. Reminders for medication, medicine purchase, and consultations were often mentioned during interviews. Although mobile phones provide reminder support, parents and persons with CF tend to prefer support integrated in a self-management app for CF. They wish to get all support for CF in a single app. Reminders are especially useful for new medication and medication that has to be taken at specific times, as illustrated in the following quotes:

For antibiotics, especially when a particular number of hours should pass between intake.

Mother of 7-year-old child

It would be good with reminders. It is true that sometimes the antibiotics treatment can be hard to follow.

Mother of 9-year-old child

It is difficult to remember unusual medication, especially when medication has to be taken outside the meals [when other medication is taken].

21-year-old adult

An app should support reminders: when and how to take drugs, when to go to the physio.

Doctor

Reminders are also useful for Creon when children start taking responsibility, or for the members of the family who do not usually follow the child tightly, as illustrated in the following quotes about reminders, with the first being on reminders for Creon:

For the child and the father!

Mother of 7-year-old

For the pancreatic enzyme. Some kind of reminder in a nice funny way. [...] When he is on holidays, when he visits the grand-parents.

Mother of 9-year-old child

#### Health Diary

A health diary supports the recording of the health status, symptoms, and other events related to the management of the disease. When discussing the influencing factors and the limitations of care, we saw that the motivation for keeping a diary is manifold. Most importantly, patients and parents expect a diary to be useful during consultations. They would better remember past events and be able to answer to the doctors’ questions more precisely. The HCPs also mentioned that parents sometimes feel embarrassed when they cannot describe symptoms. In addition to support for consultations, a diary is a means to understand the disease and the effects of the treatment. Furthermore, keeping a diary, for instance using a checklist, can provide satisfaction that the treatment is performed well, as illustrated in the following quotes:

An app which makes it possible to cross out when a task is done...There are many tasks to accomplish in a day, so it’s nice with a checklist. It’s a good feeling to see that assignments are “checked out”...One can go back and see what has been done. It’s nice for remembering afterwards what was done.

Mother of 2-year-old child

Collecting information with an app is more systematic than what you can say over the phone, or when you sit and fill out a form and then insert it in a PC.

Doctor

Patients often forget what has happened between consultations. An app for following day-by-day would allow more correct estimations. The patients could keep a diary without sending it systematically to the doctor, allowing them to answer the questions of the doctors. The diary could contain information about coughing during night, coughing during exercise, shortness of breath, stomach pains, diarrhea, problems taking pills, feeling bad. A list with ticking would make it easy. A weekly diary may be sufficient.

Doctor

A diary is most useful for parents with *little experience* with the disease or when *health conditions are changing*, as illustrated in the following quote:

We remember well [his health condition] because he is stable. I imagine that it would be nice with an agenda or something like this if he was not stable.

Mother of 17-year-old child

Keeping a dairy should be simple to handle in an otherwise busy day. One HCP stated the following:

It should be focused on things that are important to report. And it should be easy, for instance in form of a check list. Very concrete things such as “Did the child cough during the night?” “Did the child have stomach pain?” should be covered. There could also be a field for notes.

Patient association representative

#### Treatment Follow-Up

When a diary is kept, it is possible to visualize the health status over time. It is also possible to relate health to the different parts of the treatment such as enzyme management and nutrition management. Without visualization, the motivation for recording the health status is low, as illustrated in the following quote about diary:

It would have been nice, also with a space for noting changes in condition, so that one can compare over time. If not, there’s no point.

Mother of 2-year-old child

Following progress over time and effect of medication is also important for doctors. One HCP stated the following:

Anything that can increase compliance will be fine. [...] It could be useful to detect the effect of antibiotic cures and the side effects. It is important for the therapists to know how the previous cure worked when setting up a new antibiotic cure.

Doctor

It is important to support and encourage people with a serious disease, for example, through keeping focus on things that work, as illustrated in the following quote:

It is important to provide a positive educational solution. An app should put attention to what works well. And maybe a graphical overview where the patients can see how the daily life looks like and how things change. May be a score, and then they can check off [entries] throughout the day, and get points for the day. And it is important that those who are very sick also score high.

Nurse

#### Communication With Doctors

The parents and adults in the study were mostly satisfied with the current means in place for communicating with the HCPs. However, a self-management app that supports nutrition management and health diary makes it possible to offer new forms of communication. Collected data can be shared with HCPs. Parents and persons with CF in the study are willing to share data and believe sharing is positive for their health, as illustrated in the following quotes:

I would share my diary with the doctor so he could quickly see information about me. I have nothing to hide. It is best for me, when he knows more about me.

21-year-old adult

To register information and share it with doctors would be nice. It would be easier to inform the doctor.

19-year-old adult

Nonetheless, they feel that it is important that parents and patients can keep control of what is being shared. Two parents stated the following about sharing registered data:

It would have been nice, but the patient must make the choice himself and he should decide what information is shared. I believe that most patients would share if this can help them.

Mother of 2-year-old child

There is no guarantee that everyone has a good relationship with their doctor. The sharing of information must be made after agreement with your doctor.

Mother of 8-year-old child

As earlier mentioned, a doctor pointed out that noncompliant patients would probably not reveal that they do not follow the treatment.

Most HCPs would study the data during consultations, but they have no time to follow up every patient more often. They were worried that some patients would expect the data studied directly, once it is shared. Indeed, parents and persons with CF have various expectations, as illustrated in the following quotes:

It would be nice if the doctor could get one month status for example. The doctor does not have the capacity to follow up on a daily basis, but a regular overview of symptom development would have been nice.

Mother of 8-year-old child

It would be good to register things such as when I am coughing more. It would be good if the doctor can see that you have this problem now and then. It could be good to tell how you cough or what colour the mucus has. It would be good if the doctor could see more often than every three months. This would make it possible to anticipate, and maybe avoid hospitalisation.

21-year-old adult

Other concerns of the doctors are privacy and responsibility when data is being shared, as illustrated in the following quote:

You cannot drown in information. And it must be handled privately. As soon as information comes to the hospital, it is sensitive health information. You have to agree how often to register the information as well.

Doctor

Communication with doctors covers more than sharing data registered by parents and children and teenagers with CF. The setting of nutritional goals by dietitians and the sending of motivational messages by HCPs were also considered.

#### Communication With Peers

In some of the countries involved in the study, well-functioning communication channels exist where persons with CF and caregivers can get support and tutoring from peers. For instance, the CF patient association in Norway has set up two closed Facebook groups: one for all members and one for adults with CF. Both groups are very active. One parent stated the following:

We use the association a lot. The closed groups on Facebook are very important. There, we can post questions and get answers from the other members. One often gets very many answers right away. There is much happening there. It is very, very helpful.

Mother in Norway

The association has also set up a system of “peers,” as illustrated in the following quote:

The association organises a system of “peers.” It is a system where people with CF and parents can talk with other persons with CF and parents. There are 21 “peers” that have been taught through courses. They have guidelines about how to work. They are available almost the whole day.

Mother in Norway

Such channels are not available in all countries or do not function well, as illustrated in the following quotes:

Sometimes I think that it would be good to talk with somebody who can understand me, rather than to people who tell me “yes, yes.” But this is not a critical concern. I have not investigated if there exist forums. The association has a Facebook community, but there is not much happening.

21-year-old adult

We are members of the CF association. Sometimes I mail to the association. It is more when I need something. It is not active participation. On Facebook they have a page, but I think that it is not so active.

Mother of 15-year-old teenager

Digital meeting places may be well-suited communication channels for persons with CF, who should avoid meeting other persons with CF because of the risk of cross-infection. It might be challenging to keep meeting places active. It is important to define appealing activities for the participants. We saw earlier that exchanging recipes is suggested.

Some persons with CF and parents would wish for more communication with others, as illustrated in the following quotes:

Really interesting and exciting, to meet and to exchange views with other patients!

16-year-old teenager

Yes, it is good. We always help each other, giving ideas or options that may help. This would be very helpful!

Mother of 3-year-old with CF

Others wouldn’t use such a function because in their opinion, tips and tricks of one patient can’t be transferred to others, or because they suspect that false information would be spread, as illustrated in the following quotes:

Useless, because everyone is different, also as patients.

16-year-old teenager

No, too risky without a doctor supervision.

Father of 5-year-old child with CF

There are some Facebook groups, but I don’t like it, because they don’t have a clinical mediator/controller, so a lot of times other parents talk about incorrect information.

Father of 17-year-old teenager

This implies that an app or apps should give the option to communicate with others, but this shouldn’t be mandatory.

## Discussion

### Principal Findings

The findings presented here have been made possible by the overwhelming dedication of potential future users to bring forward developments that could support them in the future. The interest in the research was high, and participants openly provided their ideas and wishes. This interest and the numerous suggestions from persons with CF and caregivers indicate the readiness for self-management in the CF care even in countries that provide well-functioning health care services for CF care.

Some of the functions presented here confirm findings by previous research, such as the wish for reminders for medicine intake, a function for automated medicine refill, or the visualization of treatment progress [19]. We provide more detailed insight regarding the needs related to these functions. In addition, we identify other potentially useful functions such as support for enzyme dosage calculation and nutrition management. A recurrent need was that all functions should be included in a single app. During the search for CF-related apps performed before the interviews, such an app was not yet available on the market. The complexity with respect to the large variety of needs and related functions is one of the biggest development challenges.

An important finding is the need for personalization, stemming from the individuality of the patients, from the level of experience with the disease and treatment, and the changes in life; “One size does not fit all” in the case of CF self-management. The changing needs as children with CF grow up and gradually take responsibility for the treatment supports the idea of an ecosystem of apps proposed by MyCyFAPP. Additionally, other factors influence the care, indicating that each app in an ecosystem should be customizable. Software engineering approaches for developing adaptive software [35] and end-user development [36] are highly relevant.

The HCPs in the study failed to elicit requirements for a professional tool. Although they were asked about how a tool could facilitate the care and how a tool could fit in their workflow, they were more concerned with discussing the digital support for patients. As a starting point for the design of a professional tool, the functionalities supported by the apps can be reflected in the tool, for example, support for nutritionals goals, educational content, and treatment follow-up. In our future research, it will be important to set more emphasis on self-management as a collaboration between patients and HCPs rather than a delegation of tasks to patients. For this reason, codesign workshops and extensive testing of different versions of the prototypes together with HCPs are planned. This will allow to focus on the development of a professional Web tool covering the needs of this specific user group.

Data sharing on treatment progress with HCPs is potentially useful. Sharing can contribute to providing HCPs with a better understanding about the health status of individual patients and at the same time to increase knowledge about the disease through comparison and analysis of data collected from several patients. Several concerns should, however, be addressed. Most importantly, sharing should not be imposed on patients, but the “what?” and “how?” of sharing should be agreed on between patients and HCPs. For example, they should agree upon how often patients are expected to register data, and they should agree upon how often HCPs are expected to study the shared data. Several HCPs mentioned that the quality of data is not so good when patients are asked to register every day. This is an issue that should be further researched upon to find suiting solutions for it.

A main challenge we met in our work is the diversity of needs and the different priorities of the stakeholders. A main research topic in MyCyFAPP, the enzyme management, is a concern for parents of recently diagnosed children who need more learning support, but not necessarily for experienced parents and patients. Parents are usually more worried about nutrition than children are. Some patients, in particular teenagers, are more worried about gaining too much weight than the dietitians say they should be. HCPs wish for detailed nutritional information, but parents and patients do not wish to spend more time on their treatment, and detailed registration can be time-consuming. Parents wish to share recipes with peers, but HCPs are not always positive, as they wish to approve the advice given to patients. Some parents asked for advanced support for getting advice based on health status, but doctors warn against it. One HCP stated the following:

One should avoid giving medical advices. Advices can be dangerous without any medical supervision.

Doctor

With the growth of chronic diseases, patient empowerment, where patients manage their own health condition, has emerged as a means to release health care systems under pressure. Two important aspects of empowerment are (1) skills development and (2) choice and responsibility [37]. Choice and responsibility presume care practices that enable shared decision making (ie, the patient is involved in health care decision-making processes) and self-determination (ie, the patient has the power to choose own goals). Choice and responsibility require the patient to hold self-efficacy skills (ie, the patient is confident that he is able to carry out the necessary behavior) and self-management skills (ie, the patient has gained skills to manage lifestyle-related aspects of the disease). These skills in turn require education (ie, the patient is educated to deal with the disease and its treatment). Our study shows that a full patient empowerment is unrealistic in the case of CF. CF is a serious chronic disease that requires high expertise for decision making. The focus should be on giving patients the necessary knowledge to feel confident in carrying out the treatment.

### Strengths and Limitations

When developing apps for the therapy support of chronic diseases such as CF, which are additionally characterized by a high individuality in the manifestation of the disease, user integration in the development process is crucial. It helps not only to maximize the added value for the user but also user satisfaction [20] and ultimately influences user experience. The current research is based on a large sample of participants. It includes various perspectives by presenting the views of patients, parents, as well as HCPs. This ensures that needs and challenges of different people involved in the treatment can be considered during development. The participants who contributed to this research come from different countries across Europe and different age groups, which further increases the diversity of the sample.

Although the benefits can be large in terms of developing an app that serves actual user needs, it can be a challenging task. In this study, patients had to be interviewed one at a time to avoid cross-infection risks, so we were not able to organize group interviews allowing discussion of different viewpoints. Additionally, the views of participants represented in here reflect only those who were willing to take part in a research project. Needs or wishes by those who were not interested or prevented from participating may potentially differ significantly. Furthermore, most of the involved participants were in a more or less “stable” condition. Only very few children and teenagers with CF in a severe condition were asked to participant in the study, to not burden this user group even more. However, we were able to collect insights from HCPs, patient association representatives, and the netnography, which represent more general opinions that can partly balance out this knowledge gap. Furthermore, as participants who show interest and or are in a condition that allows them to take part in such a study are potential first adopters of the solutions, it is important to fulfil their needs.

Needs and wishes of very young patients (aged 4-7 years) mostly were collected with the help of interviews with parents and HCPs or with the help of interviews with children together with their parents. The young children involved found it very difficult to sit through an interview and give answers to questions of the interviewer, especially the more abstract these questions were. Therefore, insights regarding their needs are strongly affected by the opinions of parents and HCPs. To balance this out, this age group will be more involved later on in the project via cocreation workshops and prototype testing sessions as soon as there are more concrete products (mock-ups and prototypes) they can give feedback on.

## Abbreviations

CF: cystic fibrosis
HCP: health care professional
ICT: information and communication technology
QoL: quality of life
